# Advanced Light Metal and Alloys: Preparation, Characterization, and Applications

**DOI:** 10.3390/ma18235449

**Published:** 2025-12-03

**Authors:** Pingping Liu, Zhenyu Tian, Qian Zhan, Farong Wan

**Affiliations:** School of Materials Science and Engineering, University of Science and Technology Beijing, Beijing 100083, China

Driven by the continuous pursuit of lightweight, high-performance, and long-lasting structural materials in fields such as aerospace and transportation, research and development of advanced light metals and their alloys have become increasingly active [1]. In the transportation sector, automotive lightweighting serves as a key strategy for improving fuel economy and extending the driving range of electric vehicles. A 10% reduction in vehicle weight can enhance fuel economy by approximately 6% and increase the range of electric vehicles by about 14% [2]. Automotive lightweighting is primarily achieved through design optimization and material substitution. On the other hand, beryllium, aluminum, and their alloys are widely used in aerospace applications due to their high specific strength and excellent dimensional stability [3].

The second issue of this Special Issue [4], “Advanced Light Metal and Alloys: Preparation, Characterization, and Applications”, systematically presents the latest advancements and breakthroughs in the field through four cutting-edge research papers on aluminum alloys (the most widely used lightweight metal material) spanning compositional design, preparation processes, microstructural characterization, and performance evaluation and application [5,6,7,8]. Additionally, it includes a paper on another special lightweight metal material (beryllium and its alloys) focusing on structural and performance changes under irradiation environments [9]. These studies not only deepen our understanding of the “structure-environment-performance” relationship in materials but also provide critical technical pathways and theoretical support for the engineering application of next-generation high-performance lightweight alloys.

Traditional alloy development typically relies on macro-level adjustments of primary elements, whereas modern advanced alloy design delves into the microscopic scale, tailoring final material properties through precise control of elemental ratios and the addition of trace alloying elements [10]. Li et al. [8] investigated the influence of the Cu/Li ratio on the mechanical properties and corrosion behavior of Sc-containing Al-Cu-Li alloys. Their study revealed how the Cu/Li ratio serves as a critical design parameter by altering the matching patterns of precipitates—including their type, size, number density, and spatial distribution—thereby regulating alloy properties such as strength, plasticity, and corrosion resistance.

Advanced compositional design requires compatible preparation and processing techniques to fully realize its performance potential. Zhao et al. [5] investigated the effects of heat treatment (T4 and T6) and cold rolling on the mechanical properties and impact failure resistance of a novel Al 6082 aluminum alloy produced via the Continuous Casting Direct Rolling (CCDR) process. Their research demonstrated that combining subsequent T6 heat treatment (solution treatment + artificial aging) with moderate cold rolling (e.g., a 33.3% thickness reduction) effectively leverages the synergistic effects of precipitation strengthening and work hardening, resulting in a comprehensive performance profile with strength up to 400 MPa and plasticity meeting engineering requirements. Chen et al. [6] studied the influence of deposition parameters and deposition height on the microstructure and mechanical properties of 2319 aluminum alloy in the Laser-Cold Metal Transfer (CMT) hybrid additive manufacturing process. Their work showcased the application of Laser-CMT hybrid additive manufacturing technology in fabricating thin-walled 2319 aluminum alloy components. This technology ingeniously integrates the high energy density and precision of lasers with the high deposition efficiency and cost-effectiveness of arcs, achieving a remarkable material utilization rate of 96.43% and good forming quality. The study further revealed the gradient effects of heat accumulation and thermal cycling—induced by deposition height—on microstructure (grain size, second phase) and properties (hardness, strength, plasticity). By optimizing process parameters, samples with excellent formability and mechanical properties were obtained, demonstrating the technology’s significant potential for high material utilization and rapid manufacturing of complex aluminum alloy structural components.

In aerospace components made of aluminum and beryllium alloys, machining-induced residual stress not only influences the formulation of subsequent machining processes but also directly affects the dimensional accuracy and structural stability of final workpieces. Consequently, accurate measurement of residual stress becomes particularly crucial. Shen et al. [7] investigated the influence of rotational speed on measurement accuracy and repeatability when using the incremental hole-drilling method to determine machining-induced residual stress in aerospace aluminum alloys. The reliability of the measurement results was evaluated through finite element simulations and experimental validation (by measuring the deformation of thin-sheet samples).

The performance of materials is intrinsically linked to their service environment. For instance, beryllium and its alloys, when used in aerospace or nuclear reactor applications, are exposed to neutron or other energetic particle irradiation [11]. Liu et al. [9] fabricated beryllium-titanium and beryllium-tungsten alloys using hot isostatic pressing and conducted a comparative study on the helium-ion-irradiation-induced blistering behavior of beryllium and its alloys (Be-Ti and Be-W) under high-dose conditions. These findings provide preliminary experimental evidence for evaluating the irradiation swelling resistance of beryllium alloys. Furthermore, their research group experimentally determined, for the first time using conventional transmission electron microscopy (TEM), the displacement threshold energy (Ed) of metallic beryllium—a fundamental parameter crucial for studying irradiation damage in beryllium [12]. This achievement offers valuable reference data for both experimental and theoretical simulations of irradiation damage behavior in metallic beryllium.

The future development of this field is likely to depend on: (1) Deep integration of artificial intelligence and computational materials science to accelerate the design of new alloy compositions and processing techniques [13,14,15,16,17]; (2) Multi-process hybrid manufacturing technologies, such as combining additive manufacturing with subsequent heat treatment and plastic deformation, to achieve proactive control of microstructure and properties [10,18,19]; (3) Development of novel lightweight metal materials for extreme environments (e.g., radiation resistance in fusion reactors, high-low temperature cycling in aerospace applications) [20,21,22]. With continued breakthroughs in these directions, advanced lightweight alloys are poised to play an increasingly vital role in humanity’s quest for exploration and sustainable development.
